# Mobile Technology to Monitor and Support Health and Well-Being: Qualitative Study of Perspectives and Design Suggestions From Patients Undergoing Hematopoietic Cell Transplantation

**DOI:** 10.2196/49806

**Published:** 2023-08-31

**Authors:** Cinzia Caparso, Gwynne Ozkan, Maxwell Kluge, Humza Salim, Aidan Khaghany, Amanda Blok, Sung Won Choi

**Affiliations:** 1 Department of Systems, Populations and Leadership Center for Improving Patient and Population Health University of Michigan School of Nursing Ann Arbor, MI United States; 2 Rogel Cancer Center University of Michigan Ann Arbor, MI United States; 3 Department of Pediatrics, Blood and Bone Marrow Transplant Program University of Michigan Michigan Medicine Ann Arbor, MI United States; 4 Veterans Affairs Center for Clinical Management Research Ann Arbor Veterans Affairs Health Care System Ann Arbor, MI United States

**Keywords:** hematopoietic stem cell transplantation, bone marrow, surgery, surgical, recovery, mobile apps, mHealth, mobile health, app, apps, qualitative research, qualitative, experience, experiences, perception, perceptions, stem cell, stem cells, transplant, transplantation, transplants, hematopoietic, positive psychology, psychology

## Abstract

**Background:**

In the United States, hematopoietic stem cell transplant (HCT) surpasses 22,000 procedures annually. Due to the demanding and time-intensive process of an HCT, patients and family care partners face unique challenges involving their health and well-being. Positive psychology interventions (PPIs) may offer potential solutions to help boost health and well-being.

**Objective:**

This study aimed to explore and understand patients’ experiences and perceptions about the use of the Roadmap 2.0 app, specifically its PPI features, during the acute phase of HCT.

**Methods:**

From an ongoing randomized controlled trial, HCT patients (n=17) were recruited to participate in semistructured qualitative interviews between October 2022 and January 2023 within a large academic medical center in the Midwestern states. Using a qualitative descriptive approach, interviews were conducted in person or via Zoom. The data were analyzed through constant comparative analysis. The Consolidated Criteria for Reporting Qualitative Research (COREQ) guidelines were followed.

**Results:**

The majority of the participants reported Roadmap 2.0 as easy to use and enjoyed the ability to track their health data (eg, steps, mood, sleep; 9/17). Regarding the use of PPIs during the acute phase of treatment, 88% of the participants reported interest in participating in PPIs, specifically the Pleasant Activity Scheduling (11/17) followed by Gratitude Journaling (7/17) activities. Additionally, participants provided recommendations on adapting Roadmap 2.0. The major recommendations were (1) “Working Together: Need for Dyadic Involvement,” (2) “Connectivity with Other Patients,” and (3) “Gap in Nutritional Support.” Participants (10/17) expressed the importance of caregiver involvement in activities beyond treatment-related management for maintaining healthy patient-caregiver dyadic relationships. They also expressed their desire for connectivity with other patients undergoing HCT, primarily for comparing experiences and discussing topics such as symptom management (8/17). Lastly, participants identified a gap in nutritional support during the HCT process and expressed interest in an intervention that could promote healthy eating through education and notification reminders (9/17).

**Conclusions:**

Participants openly expressed their eagerness to participate in research studies that foster connection and positive relationships with their caregivers as well as with other HCT patients. They emphasized the significance of having access to nutritional support or guidance and highlighted the potential benefits of using mobile technology to enhance these collective efforts.

**Trial Registration:**

ClinicalTrials.gov NCT04094844; https://clinicaltrials.gov/study/NCT04094844

**International Registered Report Identifier (IRRID):**

RR2-10.2196/19288

## Introduction

In the United States, annual hematopoietic stem cell transplant (HCT) surpasses 22,000 procedures annually [1]. While HCT is a potentially curative treatment for many cancers, it has significant morbidity and mortality rates [2]. HCT is a rigorous, strenuous, and time-intensive process that impacts patients’ psychological well-being and their overall quality of life [3]. Additionally, the HCT process is so intensive that patients are required to identify at least one care partner (henceforth, this term will be used interchangeably with unpaid caregiver, eg, spouse, sibling, child, other family member, neighbor, or friend) to provide around-the-clock care for roughly 12 weeks after transplantation [4]. Due to this high-burden treatment regimen, patients as well as caregivers face their own unique set of challenges, such as a strenuous caregiving role, physical discomfort, fatigue, and psychological distress, including anxiety and depression [5,6]. Therefore, patients and caregivers report a crucial need for social support from other patients as well as other family members to assist them in maintaining positive emotions throughout the HCT process [7,8].

Positive psychology interventions (PPIs) have been developed to fill this gap [9]. PPIs deviate from the traditional “fix-what’s wrong” medical model and focus on highlighting the individuals’ strengths to increase overall well-being [9]. PPIs in cancer patient-caregivers (ie, dyads) have resulted in increased psychological well-being, dyadic coping, and decreased physical discomfort, such as fatigue or pain, for both the patient and caregiver [9]. The majority of these PPIs broadly target mainly breast and prostate cancer patient populations using in-person intervention [9]. To date, PPIs delivered using mobile technology for the HCT patient and caregiver population have been limited [10]. Due to the large burden placed on the HCT population related to treatment and follow-up regimens, technology-facilitated interventions are necessary for accessibility and management. One mobile health app for HCT patients addresses their general health, food intake, stool count, exercise, sleep habits, stress, and symptoms [11]. However, this intervention has not been tested for efficacy and does not include the caregiver as a support during treatment and recovery from HCT treatment. There is a lack of technology-delivered interventions for this psychologically vulnerable population that support dyadic interactions related to health care management as well as psychological well-being.

Therefore, our team developed a mobile-based technological intervention for the HCT population (Roadmap 2.0), which provides PPIs integrated with wearable sensors to promote caregiver well-being. This study is currently enrolling HCT patients and caregivers (NCT04094844) [10]. In the context of a 2-arm randomized controlled trial, we conducted a series of one-on-one interviews with HCT patients who received a control version of the app and whose caregivers were randomized into either intervention or control groups. We sought to explore and understand patients’ experiences and perceptions of their participation in the Roadmap 2.0 study, their views of the Roadmap 2.0 app (control version), and the potential benefit of PPIs during the acute phase of HCT, including their mental health and well-being.

## Methods

### Intervention Description

A description of the Roadmap 2.0 study has previously been published [10]. The full version of Roadmap 2.0 includes a multicomponent app that integrates with wearable sensors (eg, Fitbit Charge 3), provides a platform for patient-reported outcome data to be administered, including a daily mood questionnaire (“How has your mood been today,” 1=worst possible, 10=best possible), PPIs (savoring, pleasant activity scheduling, random acts of kindness, signature strengths, love letter, engaging with beauty, gratitude journal, and positive piggy bank activities; Table 1), and a chat forum to connect with other caregivers. The chat forum provides a platform for caregiver users to post positive comments based on the respective PPI activity exercise, which are shared with other participating caregivers. In the randomized controlled trial, caregivers are randomized to receive either the full version of the app or the control version. While patients consent and enroll in the study with their caregiver, they receive a “control” version of Roadmap 2.0 that includes graphical trends of their mood, steps, and sleep only (ie, no PPIs or chat forums). The patient and caregivers both receive wearable sensors (eg, Fitbit Charge 3) and provide patient-reported outcome responses through the app.

**Table 1:** Positive psychology intervention descriptions.

Positive psychology intervention	Description
Positive piggy bank	Users can insert happy memory entries over time, then “break open” the piggy bank to review the entries to savor the memories and remember those good feelings. Users are encouraged to insert one memory per day.
Gratitude journal	Users write what they are grateful for to inspire feelings of appreciation and optimism. Users are encouraged to write at least three entries per day.
Savoring	Users are tasked with making special moments last longer so that they become more meaningful and memorable. Users are encouraged to savor 2 activities per day.
Pleasant activity scheduling	Users can schedule time to focus on activities they enjoy, with the goal of improving their mood, reducing stress, and increasing energy. Users are encouraged to schedule one pleasant activity per day.
Random acts of kindness	Users are tasked with completing acts of kindness for others and for themselves, and then reflecting on the positive impact those actions have. Users are encouraged to pick 1 day per week to do 5 acts of kindness.
Signature strengths	Users can take a questionnaire to assess their character strengths. They will then be tasked with using their strengths in unique ways and reflecting on the impact of their actions. Users are encouraged to apply their strengths in a new way each day for 1 week.
Love letter	Users are asked to think about their loved ones (specifically those they are providing care for), write a letter to that loved one, and then share their letter with the person they wrote it to.
Engaging with beauty	Users can log the beauty they observe in their lives, both through pictures and text. The goal of this activity is to improve the user’s outlook on the world around them.

The Fitbit Charge 3 is an advanced touch screen fitness tracker that monitors variables such as heart rate, calories burned, pace, distance, and sleep cycles. The device can be worn on the wrist and wirelessly connects with the patient’s smartphone via Bluetooth to the Fitbit mobile app (available on Android and iOS systems). Participants had access to their data and, if they wanted, could set fitness goals within the app. The participants were instructed to wear the Fitbit device continuously. The smartwatch uploaded data to the participant’s phone every 15 minutes via Bluetooth Low Energy [12]. The study team had access to the patient dashboard and data download for analysis through the Roadmap app. In addition to the study team, participants also had access to view their health data in real time on their Fitbit app.

### Ethics Approval

This study was approved by the institutional review board of the University of Michigan Medical School (HUM00165192).

### Study Design, Recruitment, and Consent

The Consolidated Criteria for Reporting Qualitative Research (COREQ) was used as part of manuscript preparation. The COREQ checklist is shown in Multimedia Appendix 1. Patient-caregiver dyads were recruited from the adult and pediatric bone marrow transplant (BMT) units of a large academic medical center in Midwestern states. Research coordinators with prior recruitment experience and job training in qualitative methods purposively recruited patients who had previously consented and enrolled in the Roadmap 2.0 study. All interviews were conducted in person (n=11) or via Zoom teleconference (n=6; Zoom Video Communications) and were audio-recorded with permission. Caregivers were commonly not present during patient interviews. A total of 32 patients were approached for the study, 13 participants declined to participate, and 19 patients were recruited for the study, but 2 did not show up for their scheduled interviews. These one-time interviews ranged from 5 to 35 minutes (mean 11 min, 46 sec). Compensation was provided to all participants.

The interview guide was informed by the literature [13] as well as our previously published data, as seen in Multimedia Appendix 2 [14]. Interviews were transcribed via a transcription service (eg, BabbleType LLC). Transcripts were verified to confirm the transcription service’s accuracy. Initially, 4 transcribed interviews were individually coded by 2 coders (CC and GO) via the comments tool in Microsoft Word. The 2 coders (CC and GO) developed an initial codebook with full code labels, short code labels, code descriptions, and example quotes. A third coder (MK) applied the initial codebook to the 4 initial interviews to determine the confirmability of the codebook. The 3 coders (CC, GO, and MK) coded the data and discussed any discrepancies in the coding. A final codebook was developed and applied to the remaining interviews with 2 new coders (HS and AK). Once all interviews were coded and data saturation was met, all coders (CC, GO, MK, HS, and AK) collaborated and came to an agreement on the final themes. Key findings were derived from the data. Coding data was analyzed for the most prominent findings to determine the best ways to improve Roadmap 2.0. Transcripts were not returned to participants, and participant checking did not occur due to potentially little benefit or gain in research compared to the illness burden experienced by this population.

## Results

### Overview

As shown in Table 2, 17 HCT patients consented to the interviews. Of the 17 patients, 10 had a caregiver in the intervention group, and 7 had a caregiver in the control group. The median age of the population was 48 (range 24-68) years. The majority were White (n=14, 82%) and identified as Non-Hispanic or Latino (n=15, 88%). More than half were male (n=9, 52%) and married or in a domestic partnership (n=9, 52%).

**Table 2:** Patient demographics (N=17).

Characteristics		Participants
**Gender, n (%)**		
	Male	9 (53)
	Female	8 (47)
**Ethnicity, n (%)**		
	Hispanic or Latino	1 (6)
	Non-Hispanic or Latino	15 (88)
	Unknown	1 (6)
**Race, n (%)**		
	White	14 (82)
	Black or African American	1 (6)
	Asian	1 (6)
	Unknown	1 (6)
**Age at enrollment (years), n (%)**		
	20-29	3 (18)
	30-39	3 (18)
	40-49	3 (18)
	50-59	1 (6)
	60-69	7 (40)
**Marital status, n (%)**		
	Single, never married	4 (24)
	Married or domestic partnership	9 (52)
	Divorced	4 (24)
**Education, n (%)**		
	High school or GED^a^	4 (24)
	Some college or 2-year degree	8 (47)
	4-year college graduate	3 (18)
	More than a 4-year degree	2 (11)
**Annual income (US $), n (%)**		
	<$10,000	3 (18)
	$10,000-$14,999	2 (11)
	$25,000-$34,999	1 (6)
	$35,000-$49,999	3 (18)
	$50,000-$74,999	3 (18)
	$75,000-$99,999	1 (6)
	$100,000-$200,000	1 (6)
	Unknown	3 (18)

^a^GED: General Education Development.

Many of them were educated with at least a 2-year college degree (n=13, 76%; Table 2). Based on their experience as “control” participants, the majority of the patients reported Roadmap 2.0 as easy-to-use and enjoyed tracking their health data (eg, steps, mood, sleep) (9/17, 53%). Additionally, 4 of the 9 reported using the app, but due to cancer-related fatigue, they did not engage with it as frequently as they desired. Two of the 17 reported not using the app at all, and 6 of the 17 were asked about the app use but did not provide a response during the interview.

Almost all of the patient participants reported interest in using PPI activities during the acute phase of their HCT treatment (15/17, 88%). The 8 PPI activities that were available to caregivers during the clinical trial were explained to these patient participants during their interview process (Table 1). Out of the 8 PPI activities, the majority of the patients reported pleasant activity scheduling as an activity they would like to participate in (11/17, 64%); followed by gratitude journaling (7/17, 41%). Several participants were interested in each of the other activities, including savoring (5/17, 30%), random acts of kindness (5/17, 30%), signature strengths (5/17, 30%), love letters (3/17, 18%), positive piggy bank (3/17, 18%), and engaging with beauty (3/17, 18%). Two (12%) participants reported love letters as an activity they would not be interested in participating in.

In addition to the patient’s views and experience of Roadmap 2.0 and the PPI activities, patients also provided recommendations about adapting Roadmap 2.0 (Textbox 1). The major recommendations revealed from the qualitative data analyses included the following themes: (1) dyads working together, (2) connectivity with other patients, and (3) gaps in nutritional support.

**Identified themes (N=17).**

**Overall patient experience with Roadmap 2.0 and view of engaging in positive psychology activities**

- Positive patient experience with app: Patient’s overall view of engaging in the Roadmap 2.0 was positive (n=9)
  *I like the Roadmap app. I think it’s easy to understand and easy to use.* [24-year-old male, 44 days post transplant; bone marrow transplant (BMT) 072]
  *I did the mood and I appreciated that because it helped me keep a positive attitude.* [65-year-old female, 156 days post transplant; BMT 062]
- Interest in positive psychology activities: Almost all of the participants reported interest in using positive psychology activities during the acute phase of their hematopoietic stem cell transplant treatment, specifically pleasant activity scheduling (n=15):
  *Activities Scheduling, just do a board game, or card game, or something nice for myself.* [48-year-old female 24 days post transplant; BMT 082]
  *As far as pleasant activities, just keep me on a schedule and to actually remember to do things because otherwise, I’d probably lay in bed all day. It’s surprising how good you can feel from just getting up and doing something.* [28-year-old male 20 days post transplant; BMT 081]
  *[Patient describing Positive Activity Scheduling] Family is what pretty much surrounds… Family is everything to me.* [63-year-old male 62 days post transplant; BMT 223]
  *I think that’s something that stuck out to me was probably the more the scheduled activities, I’d say, where you can step back and do the things you like.* [24-year-old male 44 days post transplant; BMT 072]
  *[The caregiver and patient] They would be able to share things back and forth, like a signature strength, or the caregiver would be able to see what the patient had scheduled or journaled.* [35-year-old female 64 days post transplant; BMT 225]

**Patient’s recommendations for Roadmap 2.0 updates**

- Working together: need for dyadic involvement
  Reflects the participants’ views on the need for dyadic communication and activities. This includes discussing cancer related concerns and engaging in team activities (n=10)
- Connectivity with other patients
  Reflects the participants’ views on connecting with other patients to receive cancer related support. This includes support about cancer experience (eg, treatment, symptom management, psychological support; n=8)
- Gap in nutritional support
  Reflects the participants’ views on the need for nutritional education during the BMT process. This includes the dos and don’ts of what to eat, maintain a food log, and healthy recipes and recommendations (n=9)

### Working Together: Need for Dyadic Involvement

Out of 17 patients, 10 expressed the benefit of their caregiver’s (dyadic) involvement during the HCT treatment process, supported by a PPI activity. Patients desired interaction with their caregiver to be facilitated by the app and for features to communicate or provide understanding to be present within the app. Primarily, patients thought the app could promote relationship-building (ie, activities to promote working together or activities both enjoy), communication (ie, discussing self-care goals and mood together), physical activity (eg, walks), and normal life (ie, time together, time with family, “little things” like dinner and nature). Patients believed that discussing their concerns and engaging in team activities with their caregivers was essential to maintaining a healthy relationship. Patients expressed that small activities, such as going out to dinner with the family or praying together during dinner time, strengthen the bond between the caregiver and the patient.

We do stuff together. We went out to dinner Sunday night and spent some time with family.

63-year-old male, 62 days post transplant; BMT 223

We usually pray on it every day at dinnertime… We’re pretty much together on all that stuff. We take in walks, we love nature.

65-year-old male, 6 days post transplant; BMT 79

Working together. Team activities.

60-year-old female, 62 days post transplant; BMT 222

Secondarily, participants desired functionality to support dyadic relationships, including sharing of PPI activities, schedules, journal entries, activity within the app, text chat, and notifications to prompt conversation. Additionally, the participants reported integrating activities the patient and caregiver dyad could do together through the app that encouraged a dialogue about the patient’s caregiver concerns and strategies to discuss these issues.

It might be beneficial for other people if they don’t have as good a relationship, if they’re struggling with how to talk to their caregiver about what they really need kind of thing, or if they can be more supported.

37-year-old female, 64 days post transplant; BMT 75

They would be able to share things back and forth, like a signature strength, or the caregiver would be able to see what the patient had scheduled or journaled. Maybe the patient couldn’t see what the caregiver did, but the caregiver could see what the patient was doing within the app.

35-year-old female, 64 days post transplant; BMT 225

### Connectivity With Other Patients

Out of 17 patients, 8 expressed their desire to connect with other patients going through the cancer experience and chemotherapy to receive timely and greater amounts of support. They wished to discuss their cancer experience, ranging from treatment to symptom management to psychological support, with other patients.

Chatting with others to see how their journey is going and see, are they struggling with the same things I’m struggling with? Yes, I believe that the chat forum would definitely be a positive thing.

35-year-old female, 64 days post transplant; BMT 225

Absolutely…Other patients, people that are going through similar stuff that you’re going through…

63-year-old male, 62 days post transplant; BMT 223

Overall, patients expressed a positive attitude toward connecting with other patients to compare and share struggles with fellow cancer patients.

### Gap in Nutritional Support

One of the aspects of cancer treatment that is not emphasized enough is nutrition. Participants expressed their concerns about a gap in nutritional support, as they wanted to know what was permissible and what was not during the HCT process. Out of 17 participants, 9 expressed their views on the lack of nutritional support while in-patient and at home. The transition process from in-patient to home impacted the confidence participants placed in their nutritional support. While in-patient, participants knew what they were eating every day, but they were not confident in their nutritional decisions. Participants would like common answers health care professionals would provide around nutrition questions patients have, as well as a food log or journal to support reporting to providers what they are eating. Patients desired knowledge on what foods to avoid during treatment as well as what foods and eating schedules were best during the treatment process while in-patient and at home. What to cook and recipes with financial constraints are desired.

I had to start making my own journal and writing down what I would eat, so when the nurse would come in halfway through the day and she’d say, “What did you eat?” and I’d be like, “Oh.” I’d try to remember, but I would write it down, so then I’d say, “Okay, I had this, this and that” that I had ordered, but if I could just do it on my phone, that would be nice.

40-year-old female, 145 days post transplant; BMT 64

Nutrition. I think that’s one area that we’ve struggled with the most, and not very many people know very much.

69-year-old male, 6 days post transplant; BMT 79

…what we could eat and make sure that we’re eating properly and at the right time. Stay away from certain foods, which is… No money, that means no going out, so we have to make food.

60-year-old female, 62 days post transplant; BMT 222

I think dietary stuff would be helpful, like maybe it being able to ask dietary stuff within the app.

35-year-old female, 64 days post transplant; BMT 225

…the diet one would be a good idea…in the beginning there were certain things they didn’t want me to eat.

66-year-old female, 68 days post transplant; BMT 77

Patients suggested that Roadmap 2.0 incorporate food logs, have access to healthy recipes, and gain more access to dietary information.

## Discussion

### Principal Findings

In our qualitative descriptive study, we explored patient experience and perceptions of a technology-delivered PPI that integrated with wearables. Understanding these perspectives has implications for intervention design for this population and an improvement on the current design of Roadmap 2.0. The majority of the HCT participants enjoyed participating in the study and engaging with Roadmap 2.0. Importantly, they reported interest in interacting with the PPI activities. We identified 3 main findings of desired functionality for a technology-delivered intervention for the HCT patient population. First, HCT patients expressed the critical importance of interacting with technology as a dyad. This was essential for building and maintaining a healthy relationship. Secondly, HCT patients would like connectivity through technology with other patients undergoing HCT treatment to compare experiences and discuss or hear about a range of topics, such as symptom management. Third, patients identified a gap in nutritional support in their cancer care, which technology could address by providing educational resources. We place these findings in the context of the literature and future directions.

### Comparison to Prior Work

#### Dyadic Relationship and Involvement

Our participants discussed the need to engage in positive activities with their caregivers, but they also elaborated on the need to have assistance in communicating cancer-related concerns to the caregiver. The concerns were not only the patient communicating their symptoms to their caregiver but also engaging the caregiver about how the patient’s cancer was impacting the caregiver, both physically and mentally. Patients and caregivers have an undeniable interdependence during the cancer trajectory [5,15]. The dyadic relationship can negatively impact the quality of life and psychological distress for both the patient and the caregivers [15]. The majority of our participants reported pleasant activity scheduling as the most common PPI activity they would like to engage with. The participants focused on the importance of carving time out of the day to participate in activities that brought them joy, not only for themselves but for their caregivers as well.

Haase and colleagues’ [16] therapeutic music video intervention impacted the resiliency of adolescent and young adult HCT participants by proving an intervention that allowed participants to interact with family and friends via music videos to share their experiences, foster meaningful conversations, overcome difficulties, and express gratitude to their loved ones, which impacted the patient’s self-reported outcomes (eg, positive coping, social support, and family function). While Haase and colleagues [16] focused on the adolescent and young adult HCT population, Polomeni and colleagues [17] provided a different lens for an older HCT population. In their mixed methods study, they found a disconnect between patients and caregivers due to the impact of cancer on their social relationships. These limited studies suggest the need for further development of novel dyadic interventions.

#### Connectivity With Other Patients

Hematopoietic stem cell transplantation is an intensive treatment, not only physically but mentally, as patients follow strict protocols and procedures, which cause them to report increased anxiety, depression, isolation, and sadness [5]. Patients are aware of the caregiving load that comes with the HCT process, where they engage in “protective buffering” to shield their caregivers from cancer-related thoughts or concerns to avoid adding additional burden to their caregivers [3,4]. As such, it is natural for patients to want a support system outside of their caregivers. HCT participants have reported a lack of peer connection in other interventional studies as a limitation [17]. Previous research supports that increasing peer connection allowed HCT patients to compare their experience with others and gain a sense of others understanding their experience, which put the cancer experience in perspective for participants (eg, compared to other people, I’m doing well) [17]. Accordingly, our findings suggest that patients, in addition to discussing cancer-related concerns with other patients, would also like to connect with other patients who may understand the experience of HCT and can provide suggestions or recommendations to each other.

#### Nutritional Support

Cancer treatment–related side effects, such as nausea, a decrease in appetite, and being immunocompromised, are common in the general cancer population, where the nutritional support goal is to maintain a healthy weight [18]. The importance of nutritional support in HCT patients is prevalent in Pulewka and colleagues’ [19] cross-sectional study, where 96% of their study population reported a moderate or poor nutritional intake after cancer treatment. Our participants reported needing support in deciding what to eat during the HCT trajectory to decrease the risk of infection due to their prolonged immunocompromised states. Providing informational support has been identified as an approach to reducing stress and anxiety [20]. To explore the generalizability of these findings to blood cancer patients who may not qualify for a HCT due to a lack of disease severity, we recruited 2 dyads, one with B-cell lymphoma and one with acute myeloid leukemia that met our eligibility criteria. The 2 participants did not report on the need for nutritional support but did report the importance of dyadic involvement and connecting with patients. This finding suggests two things: (1) the importance HCT patients place on nutritional support during their hospital stay but also after discharge and (2) how Roadmap 2.0 may be applicable to other patients with hematological malignancies who may not qualify for HCT but still would like support in connecting with their caregivers and other patients during their own cancer experience.

### Strengths and Limitations

Major strengths of the study include a research team with extensive knowledge of the HCT population, the inclusion of qualitative researchers whose work focuses on communication within the family unit in patients with a general cancer diagnosis, and the methodological rigor applied to analyzing the data.

We recognize the limitations of our work. Our homogenous sample, primarily White men, potentially limited the range of experience and feedback we captured. It is also possible we selected a biased sample of individuals who enjoyed participation in a technology-mediated study and who experienced positive interactions with their caregivers. Our study was conducted at an academic medical center in the Midwestern States, which limits the generalizability of our findings.

### Future Research

Past interventional work conducted in this population focuses so much of the intervention work on the patient alone or caregiver alone, in addition to being conducted in person by a facilitator. Our findings suggest HCT patients want connection and positive relationships with their caregivers and other HCT patients, as well as nutritional support, using mobile technology. Future researchers should focus PPI interventions on being inclusive of the patient and caregiver and providing health care–level education (eg, nutrition) using mobile app technology.

## Appendix

Multimedia Appendix 1 Consolidated Criteria for Reporting Qualitative Studies (COREQ) 32-item checklist.

Multimedia Appendix 2 Interview guide.

## Abbreviations

BMT: bone marrow transplant
COREQ: Consolidated Criteria for Reporting Qualitative Research
HCT: hematopoietic stem cell transplant
PPI: positive psychology intervention

## Data Availability

The interview guide is made available as Multimedia Appendix 2; however, additional data may be requested from the first author.
