# Supplementary material for: Mobile Technology to Monitor and Support Health and Well-Being: Qualitative Study of Perspectives and Design Suggestions From Patients Undergoing Hematopoietic Cell Transplantation
Source: JMIR Form Res. 2023 Aug 31;7:e49806. doi: 10.2196/49806 (PMC10502589; doi:10.2196/49806)
Supplement: Multimedia Appendix 2 [file formative_v7i1e49806_app2.docx]

*Multimedia Appendix II: Interview guide*

1. What is your current understanding/impression of the Roadmap app?
   1. From your experience, what did you observe from your caregiver in relation to this application?
2. Did you ever explore the app that your caregiver was instructed on?
   1. Did the positive psychological interventions ever interest you?
   2. Which portion of the app seems the most appealing to you?
   3. What portions of the app are intuitive? Which are confusing?
   4. What functions do you anticipate would be helpful and/or relieve burdens/stress?
3. How often do you foresee yourself having used or using the application in its current model?
4. Is the Chat Forum feature appealing to you?
   1. If yes,
      1. Who would you like to be connected with? (e.g. your caregiver, other patients, other caregivers)
      2. Do you like the anonymity feature, or would you like to use your identity?
      3. Would you be inclined to post pictures or video content?
   2. If no,
      1. Why not?
      2. What instead would be appealing?
5. Do you consider the design (colors, pictures/icons, style, etc.) of Roadmap to be fresh or dated?
6. Thinking about the future…
   1. If you could create your own app for BMT patients and caregivers, what features would you include?
      1. Which of these features do you consider most practical/most impactful?
   2. Are there any existing apps you are aware of that serve a similar purpose?
   3. Is there an activity or habit that you have discovered in your own personal experience that has helped you and may help others?
   4. What is an aspect of your healthcare experience that you think goes unnoticed or gets neglected? Is there any form of relief you could imagine?
